# Supplementary material for: The Construction and Exploration of a Comprehensive MicroRNA Centered Regulatory Network in Foxtail Millet (Setaria italica L.)
Source: Front Plant Sci. 2022 May 6;13:848474. doi: 10.3389/fpls.2022.848474 (PMC9121102; doi:10.3389/fpls.2022.848474)
Supplement: Supplementary file 6 [file Data_Sheet_6.zip › Sit-miR172b_Seita.9G512600.1_176_TPlot.pdf]

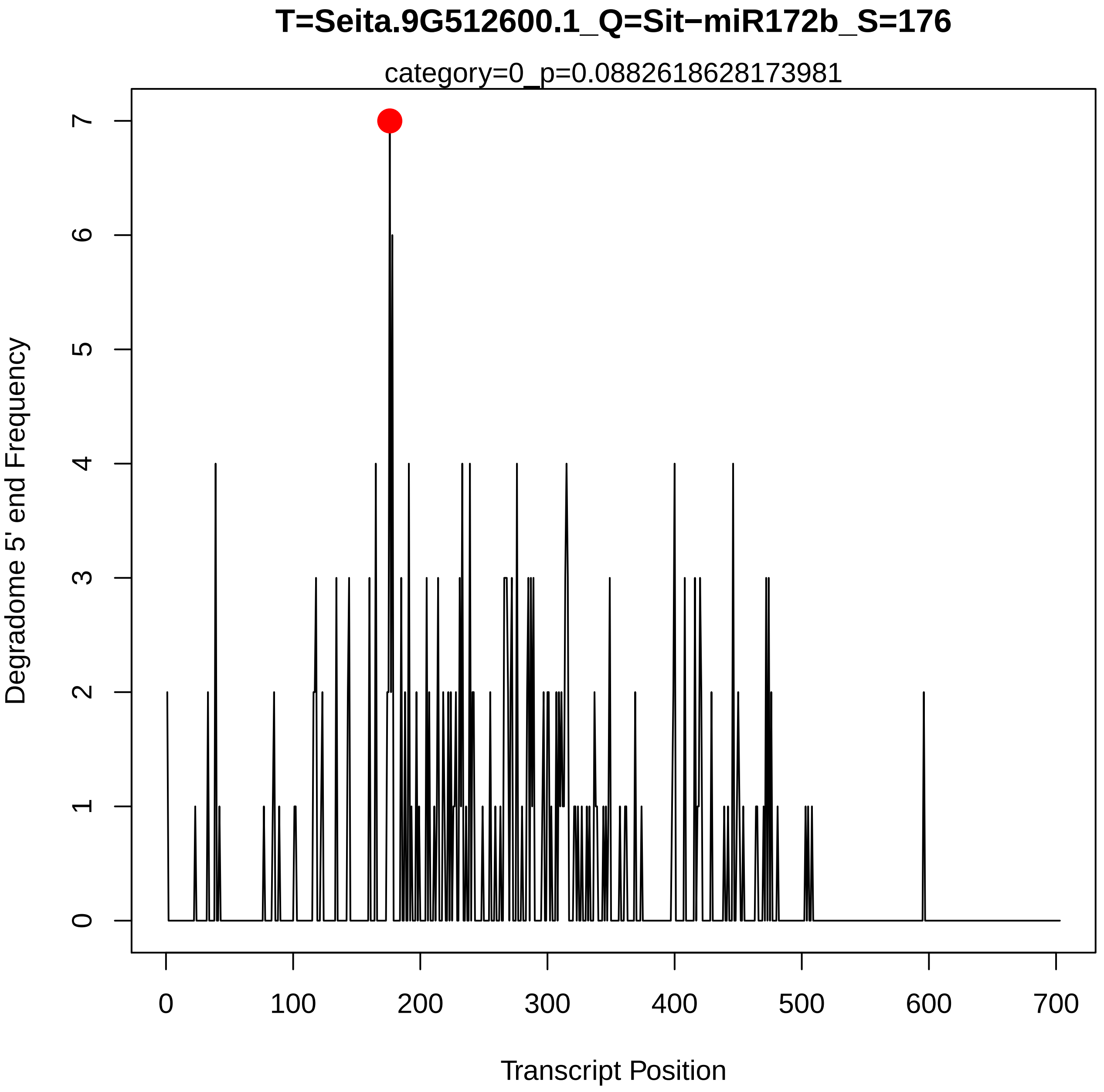

T=Seita.9G512600.1_Q=Sit−miR172b_S=176
category=0_p=0.0882618628173981
Degradome 5' end Frequency
0
1
2
3
4
5
6
7
0
100
200
300
400
500
600
700
Transcript Position
